# Establishment of endomycorrhizal fungi on micropropagated teak (*Tectona grandis* L.f.)

**DOI:** 10.1186/1753-6561-5-S7-P149

**Published:** 2011-09-13

**Authors:** Maria Isabel Ramírez Caro, Ian Bennett, Nicholas Malajczuk

**Affiliations:** 1 School of natural Sciences, Edith Cowan University, 270 Joondalup Drive, Joondalup, 6027 , Western Australia; 2 School of natural Sciences, Edith Cowan University, 270 Joondalup Drive, Joondalup, Western Australia; 3 Mycorrhizal Applications International (Australia) Pty Ltd. PO Box 1046. Bunbury, 6231, Western Australia

## Background

Commercial micropropagation of teak (*Tectona grandis*L.f.) has been achieved for a number of years in countries such as Thailand, India and Malaysia. This has led to the availability of elite genotypes for large scale plantation production. Teak has been shown to develop arbuscular mycorrhizal (AM) fungal associations [1], and this has been attributed to increasing productivity [2]. It is likely that the establishment of mycorrhiza, through the introduction of *Glomus*species, on the roots of micropropagated plantlets will improve the productivity of selected clones. Established hyphal networks can speed up plant colonization [3] and in the long term may also increase carbon storage through glomalin production [4].Our research is pursuing the development of a mycorrhization protocol for micropropagated teak at the acclimatization stage.

## Methods

Shoots of two clones of teak were multiplied on media containing Murashige and Skoog [5] nutrients and organics, 30 gL^-1^ sucrose, 0.5 µM benzyl amino purine, 0.5 µM kinetin, 2.5 gL^-1^ agar, 2.5 gL^-1^ gelitre and a pH of 5.8. Individual shoots were subsequently exposed to a medium containing ¼ strength M&S macronutrients, full strength M&S micronutrients, 60 mM sucrose, 2.5 gL^-1^ of agar and 2.5 gL^-1^ of gelrite [6], and a range of indole butyric acid (IBA) concentrations (0-160 µM) for varying lengths of time (4-28 days) to produce roots *in vitro.*Rooted plantlets were transferred to three pasteurised soil types (1sand:1perlite;1sand:1peat;and 1sand:1perlite:1peat) under the following conditions: they were maintained under mist (covered 75-95% humidity) for 5 weeks followed by hardening through gradual reduction in humidity (50-65 %) on greenhouse benches for a further 5 weeks. Plant survival, height and root area were measured.

Two clones of *T.grandis*were exposed to different inoculum sources and various inoculation techniques during the acclimatization period. Unprocessed commercial AM inoculum 100 g kg^-1^ from a legume/grass pot culture (chopped mycorrhizal roots and soil) or processed commercial AM inoculum 10 g kg^-1^ were both mixed with pasteurised inert soil (1sand:1perlite) and watered to field capacity with ½ strength sorghum nutrient solution [7]. When the processed inoculum was used, roots were also dipped into the inoculum powder before being planted. All plants were acclimatised under high humidity as above and periodically fertilized with ½ strength sorghum nutrient solution. Plantlet height, root area and mycorrhizal development were assessed at 10 and 20 weeks and means compared using analysis of variance and Tukey's multiple range test.

## Results and discussion

Optimum root production, (8.6 ± 0.7 roots per shoot) and tallest plants were obtained from shoots exposed to 80 µM IBA for 8 days. However, shoots from higher IBA (160 µM) concentrations had lower survival rates but there was no effect on the growth of the plantlets that survived. Soil type used for acclimatisation did not affect plant height and root area measurements. This allowed the acclimatisation to be conducted in a soil type that is considered most appropriate for mycorrhization development [7].

**Figure 1:**
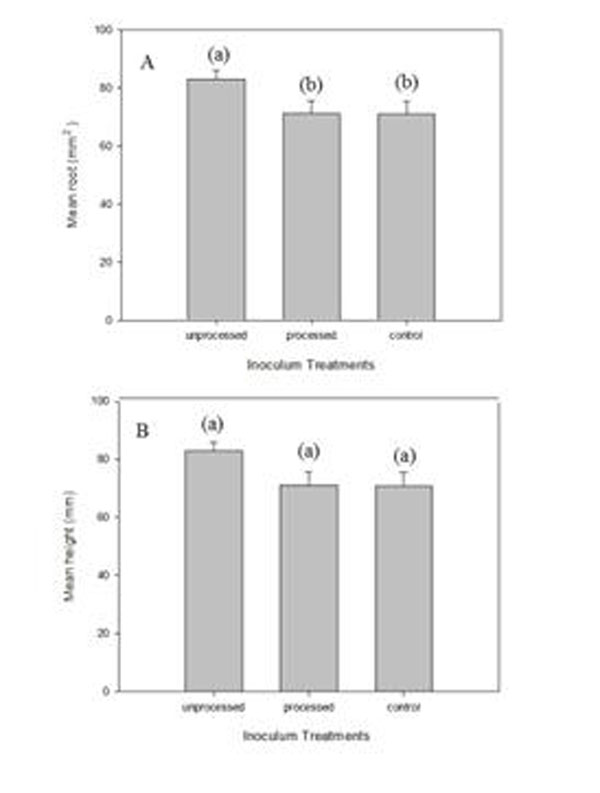
A) Mean root area and B) mean height of a single teak clone inoculated with: unprocessed AM commercial inoculum, processed commercial inoculum and control, 10 weeks after inoculation. Different letters above bars represent differences (P<0.05); error bars= standard errors.

**Figure 2:**
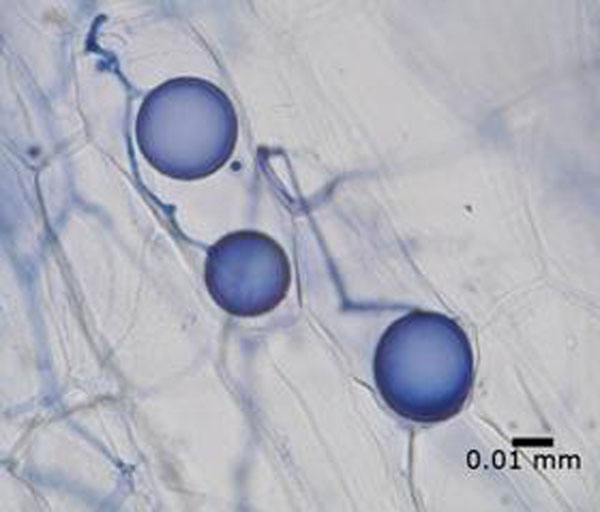
Glomoid mycorrhiza in a cleared teak root and stained with trypan blue, (X400) scale bar = 0.01 mm

At 10 weeks, 100% of inoculated plantlets had survived. Mycorrhizal inoculation using unprocessed AM commercial inoculum increased root area (609.9 ± 46.2 mm^2^; Fig 1 A & B), a similar result to that of [8] for micropropagated grapes. This was achieved, however, for one clone only. Teak root infection was also achieved using the high concentration of the unprocessed AM commercial inoculum 20 weeks after inoculation. Control plants were not infected. A preliminary comparison of the inocula used showed that early evidence of mycorrhization can also be achieved using soil pot culture (Figure 2) which agrees with [3].

## Conclusions

*T. grandis*can be inoculated and *exvitro*mycorrhization achieved during the acclimatization phase of micropropagation. The time required for the mycorrhization appears to be dependent upon inoculum source, and perhaps other factors that are currently being investigated such as inoculum concentration and substrate phosphorus contents. In addition, early results indicate that successful mycorrhization may vary between host genotypes.

The availability of clonal, mycorrhizal teak may lead to greater plantation sustainability through more appropriate fertilizer regimes and greater carbon storage.
